# The response of ecological security to land use change in east and west subtropical China

**DOI:** 10.1371/journal.pone.0294462

**Published:** 2023-11-16

**Authors:** Mengjia Luo, Jinliang Wang, Jie Li, Jinming Sha, Suling He, Lanfang Liu, Eldar Kurbanov, Janie Cole, Yuanmei Jiao, Jingchun Zhou

**Affiliations:** 1 Faculty of Geography, Yunnan Normal University, Kunming, China; 2 Key Laboratory of Resources and Environmental Remote Sensing for Universities in Yunnan, Kunming, China; 3 Remote Sensing Research Laboratory, Center for Geospatial Information Engineering and Technology of Yunnan Province, Kunming, China; 4 College of Geographical Science, Fujian Normal University, Fuzhou, China; 5 Center for Sustainable Forest Management and Remote Sensing, Volga State University of Technology, Yoshkar-Ola, Russia; 6 Council for Geoscience, Pretoria, South Africa; University of Strathclyde, UNITED KINGDOM

## Abstract

Regional land use change and ecological security have received considerable attention in recent years. The rapid economic development of Kunming and Fuzhou has resulted in environmental damage such as water pollution and urban heat island effect. It is thus important to conduct a comparative analysis of the ecological security response to land use/land cover change (LUCC) in different natural zones. Using the Google Earth Engine (GEE) platform, random forest and support vector machine methods were used to classify land cover types in the study area, after which the ArcGIS platform was used to analyze LUCC. The driving force-pressure-state-impact-response (DPSIR) model and entropy weight method were used to construct an ecological security evaluation system, and gray correlation was used to compare the ecological security responses to LUCC in Kunming and Fuzhou. The findings revealed that: (1) The average dynamic degrees of comprehensive land use in Kunming and Fuzhou from 1995 to 2020 were 1.05% and 0.55%, respectively; (2) From 1995 to 2020, the ecological security index values for Kunming and Fuzhou increased from 0.42 to 0.52 and from 0.36 to 0.68, respectively, indicating that Fuzhou’s index is rising more rapidly; and (3)There is a strong correlation between LUCC and ecological security, the correlation between the woodland and the ecological security index is very strong in both places. The expansion of construction land may be an important reason for the reduced ecological security level in Fuzhou City, while water resources have a significant impact on the ecological security level of Kunming City.

## 1. Introduction

Land Use/Land Cover Change (LUCC) represents changes to natural and anthropogenic properties of the earth surface and LUCC possesses complex spatiotemporal characteristics as well as social attributes associated with human society [1]. Ecological security refers to the health and integrity of an ecosystem, as well as its capacity to provide ecological goods and services that are beneficial to humans [2]. It is known that LUCC and ecological security have interacting effects [3]. A number of aspects of terrestrial ecosystems, including biodiversity, climate, water resources, soil condition, and landscape pattern, are influenced by LUCC. Furthermore, increasing demands for natural resources as a result of socioeconomic development have prompted major changes in land-use patterns that have a negative impact on ecosystem services [4]. Therefore, inappropriate land use endangers regional ecological security and hinders the region’s capacity for sustainable development [5].

The relationship between ecological security and LUCC has become more significant as a result of global change. Monitoring, simulation, and prediction of LUCC have become increasingly important in achieving sustainable development [6]. The pressure-state-response (PSR) model [7], the minimum cumulative resistance (MCR) model [8], the driving force-pressure-state-influence-response (DPSIR) model [9,10], the threat-quality-regulation (TQR) model [11], and the economy-environment-society (EES) model [12] have all been used in previous studies to construct index-based assessments of ecological security. The status of regional ecological security has been analyzed and evaluated in previous studies using the entropy weight method [13], the Technique for Order of Preference by Similarity to the Ideal Solution (TOPSIS) method [14], gray relational analysis [15], fuzzy matter-element analysis [16], and the landscape ecological security model [17]. There has been a gradual shift from qualitative to quantitative research on the effects of LUCC on ecological security. The theory [18], driving forces [19], and temporal and spatial changes [20] of the impacts of LUCC on ecological security have been the main subjects of these studies. The majority of these studies were carried out in cities with fast economic growth and regions with relatively fragile ecological environments [21–23]. In contrast, no comparisons of these relationships between subtropical east and west China have been conducted.Fuzhou and Kunming are located in the eastern and western subtropics of China, respectively. The natural environment and socio-economic and cultural backgrounds of the two places are quite different.Therefore, the establishment of a systematic evaluation model with unified standards and applicable to the two places to evaluate the ecological security of different natural zones can improve both the accuracy and objectivity of the evaluation results. The development of 3S technology and advancements in research methods have enabled researchers to integrate data from multiple sources [24], construct models from multiple perspectives [25], and employ a variety of analysis methods to investigate the relationship between regional LUCC and regional ecological security [26,27]. There have been numerous related studies, but the large-scale investigation has been hampered by their frequent resource and data access limitations. In light of this, the purpose of the current study was to compare the responses of ecological security to LUCC in various natural zones in Kunming and Fuzhou. Using the Google Earth Engine (GEE) cloud computing platform to complete the land use classification, then a DPSIR model was constructed to evaluate regional ecological security. The response of ecological security to LUCC in the two provincial capital cities in subtropical east and west China, which have significant differences in their natural environment, socioeconomic development, and cultural development, was characterized using quantitative and qualitative analysis techniques. The outcomes of this study can serve as a guide for maintaining ecological security in various geographical areas.

## 2. Materials and methods

### 2.1 Study areas

**Fig 1:**
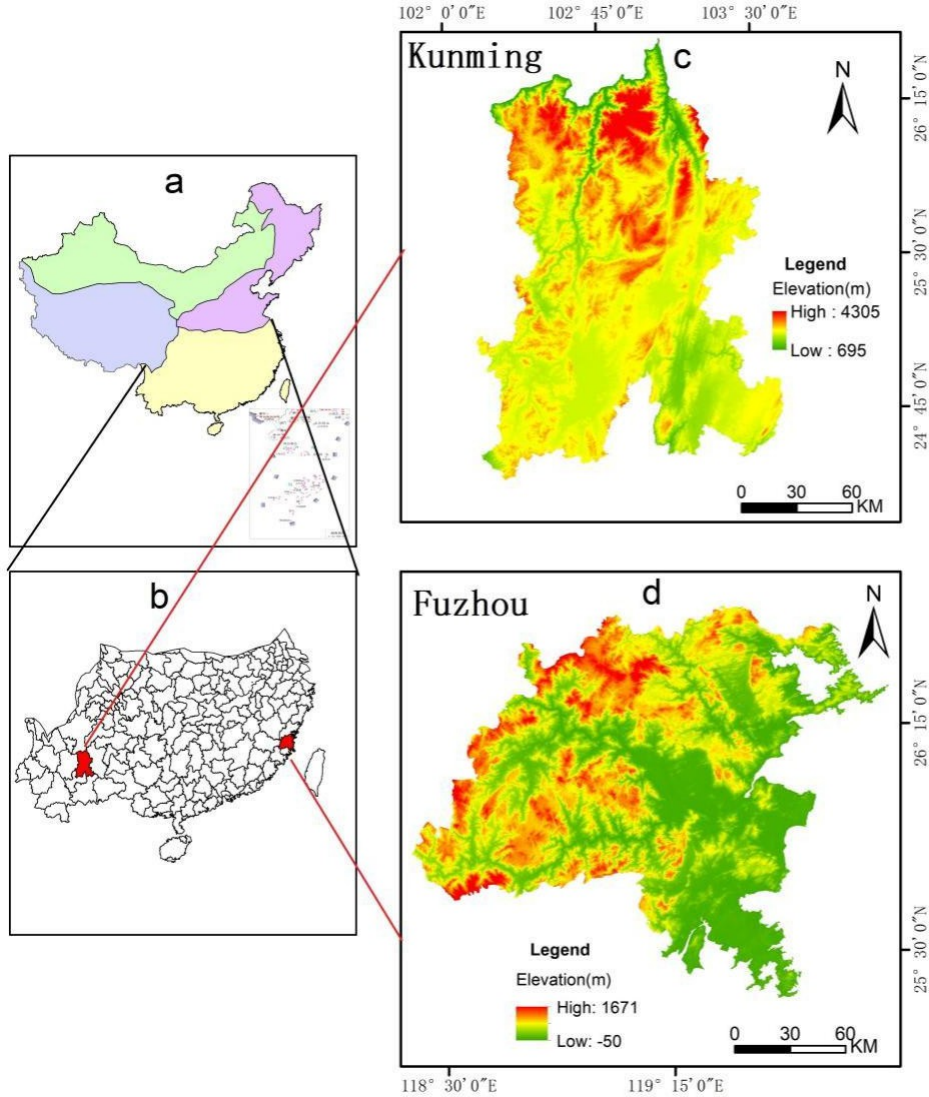
Locations of Kunming and Fuzhou. (a) climate zone map of Mainland China; (b) Relative positions of Kunming and Fuzhou; (c) Elevation of Kunming; (d) Elevation of Fuzhou.

Kunming is a major city in central Yunnan Province, located on the central Yunnan-Guizhou Plateau (102°10′–103°40′E, 24°23′–26°22′N) (Fig 1). The primary land uses in Kunming, which has an area of 21,473 km^2^, are woodland, grassland, and arable land. The climate zone of the region is subtropical mountain plateau monsoon. Kunming serves as a significant ecological buffer zone in the upper reaches of the Yangtze River Economic Belt and is characterized by an abundance of ecological resources and robust ecological service functions. The per-capita gross domestic product (GDP) of Kunming has increased by 84,667 Yuan over the past 25 years, while the city’s population density has increased by 157 people/km^2^ [28]. Rapid urban development, frequent economic activities, and increased development of the area surrounding Dianchi Lake have contributed to an increasingly complex land use pattern together with damage to the ecological environment.

Fuzhou is one of the economic zone cities on the west coast of the Taiwan Strait on the southeastern edge of the Eurasian continent (118°08′–120°31′ E, 25°15′–26°39′ N (Fig 1). Covering a total area of 11,968 km^2^, the city primarily consists of arable land and woodland. The city is located in a subtropical marine monsoon climate zone with an average annual temperature of 20 to 25°C and an annual precipitation of 900 to 2100 mm. Fuzhou’s coastal location provides many several natural benefits, including an abundance of water resources, fisheries, and remarkable biodiversity. However, due to its location in a basin surrounded by mountains, air convection within the city is impeded, leading to the urban heat island effect and coastal sandstorms during droughts. Over the past 25 years, the per-capita GDP of Fuzhou has increased by 112,796 Yuan, whereas the population density of the city has increased by 3,488 people/km^2^ [29]. The city has experienced significant increases in economic development, increases in population density, and urbanization, all of which have contributed to increased challenges,such as urban environmental pollution and unsustainable development.

While both Kunming and Fuzhou are located in a subtropical monsoon climate zone, Kunming is an inland city while Fuzhou is a coastal city. Even though both Kunming and Fuzhou play significant roles in the regional economy, Fuzhou has experienced more growth than Kunming. Land use patterns in the two cities are also distinct due to differences in topography, soil, and vegetation. Consequently, these differences in land use types between the two cities have distinct effects on the ecological security of the region. Therefore, it is crucial to characterize the ecological security responses to LUCC in various regions. This information can facilitate sustainable development coordination.

### 2.2 Data sources

The data used in the present study included socioeconomic data, remote sensing data, and basic geographic data.All remote sensing data were obtained from the GEE platform,where Landsat 5 TM data was used from 1995 to 2010, and Landsat 8 OLI data was used in 2015 and 2020. The GEE platform contains a vast quantity of global-scale Earth science data and is capable of processing large-scale data rapidly [30]. Therefore, each issue of remote sensing images is automatically screened by the GEE platform for images with less than 20% of the cloud cover in each year and then fused and cropped. The socioeconomic data were collected from statistical yearbooks and official annual bulletins, except for some data that was calculated. The basic geographical data included administrative boundary vectors for Kunming and Fuzhou. The specific parameters and data sources used in the current study are displayed in detail in Table 1.

**Table 1:** The specific parameters and data sources used in the current study.

Data type	Data name	Specification	Data source
**Remote sensing data**	USGS Landsat 5 TM	spatial resolution 30m	USGS https://www.usgs.gov/
	USGS Landsat 8 OLI	spatial resolution 30m	USGS https://www.usgs.gov/
	NASA NASADEM Digital	spatial resolution 30m	NASA https://earthdata.nasa.gov/
**Basic geographic data**	2015 vector borders of Chinese municipal administrative regions	vector data	Resource and Environmental Science and Data Center, Chinese Academy of Sciences https://www.resdc.cn/
**Socioeconomic data**	Socioeconomic Statistical Yearbook	annual data	China’s economic and social big data research platform https://data.cnki.net/
	Kunming Annual Environmental Quality Status Bulletin	annual data	Kunming Ecological Environment Bureau http://sthjj.km.gov.cn/
	Fuzhou National Economic and Social Development Statistical Bulletin	annual data	Fuzhou Statistics Bureau http://tjj.fuzhou.gov.cn/

### 2.3 Methods

#### 2.3.1 Methods for classifying remote-sensed land use

To obtain a time series of images with low cloud cover from January 1 to December 31, the present study automatically screened, fused, and cropped remote-sensed images using the Simple Composite algorithm in GEE API programming. The classification of land use types was based on previous research and the actual situation of the research area, and it was divided into six categories [31]: (1) construction land; (2) woodland; (3) water bodies; (4) arable land; (5) grassland; and (6) unused land. Extracting the normalized difference building index (NDBI), the normalized difference vegetation index (NDVI), and the normalized difference water index (NDWI) from the preprocessed data as spectral features. Extracting the slope, slope aspect, and altitude from DEM data as were used as topographic features. Spectral features and topographic features were utilized to construct a set of classification features to improve classification accuracy. The land use classification of the study area was then completed using the support vector machine (SVM) [32,33] and random forest (RF) [34,35] techniques. The classification accuracy was validated by re-selecting a set of sample points on the high-resolution Google Earth image and comparing accuracies in ENVI 5.3. The results demonstrated that the classification achieved an overall accuracy of more than 88% and a Kappa coefficient of more than 0.84, thereby meeting the requirements for accuracy. Under identical conditions of sample points and distribution range, the classification accuracy of RF was superior to that of SVM (Table 2). Consequently, the results of the RF classification were utilized for further analysis.

**Table 2:** A comparison of the classification accuracy of SVM and RF for remotely sensed images of land use.

year	Kunming				Fuzhou			
	support vector machines		random forest		support vector machines		random forest	
	overall accuracy %	kappa	overall accuracy %	kappa	overall accuracy %	kappa	overall accuracy %	kappa
**1995**	94.52	0.9288	97.26	0.9641	92.41	0.8853	92.41	0.8846
**2000**	95.89	0.9461	95.89	0.9462	89.65	0.8449	88.97	0.8831
**2005**	95.89	0.9465	94.52	0.9282	92.41	0.8871	94.49	0.9168
**2010**	89.04	0.8565	89.04	0.8571	91.03	0.8691	96.55	0.9481
**2015**	88.35	0.8475	91.78	0.8925	95.17	0.9279	95.17	0.9279
**2020**	89.04	0.8568	91.35	0.8884	92.41	0.8862	91.03	0.8640

#### 2.3.2 Analysis methods for land use change

The land use dynamic degree expresses the real and dynamic changes in different land use types. The single dynamic degree and comprehensive dynamic degree represent the degree of change of a single type of land quantity and all types of land quantity, respectively, throughout a study period [36]. Analyzing the long-term land use dynamics in a particular region can reveal the effects of human activities on LUCC and be utilized to simulate LUCC changes. Single land use dynamic degree can be calculated with the following formula [37]:

$$\text{K}=\frac{{\text{U}}_{\text{b}}-{\text{U}}_{\text{a}}}{{\text{U}}_{\text{a}}}\times \frac{1}{\text{T}}\times 100\text{\%,}$$ (1)

where *U*_*a*_ is the area of a certain type of land use at the beginning of the study period; *U*_*b*_ is the area at the end of the study period; *T* is the length of the study (years in the current study).

The dynamic degree of comprehensive land use can be calculated as follows:

$$\text{LC}=\left[\frac{\sum_{\text{i=1}}^{\text{n}}{\text{LU}}_{\text{i}-\text{j}}}{2\sum_{\text{i=1}}^{\text{n}}{\text{LU}}_{\text{i}}}\right]\times \frac{1}{\text{T}}\times 100\text{\%,}$$ (2)

where *LU*_*i-j*_ is the absolute value of the transfer area; *LU*_*I*_ *i*s the area of the i-type land use at the beginning of the research period; and *T* is the research duration.

#### 2.3.3 Method for assessing ecological security using the DPSIR model

The DPSIR model is a conceptual model that combines the benefits of the pressure-state-response (PSR) and driving force-state-response (DSR) models. The DPSIR considers all aspects of nature, economy, and society, and possesses the qualities of integrity and flexibility [38].

### (1) Establishment of evaluation indicators

The selection of evaluation indicators is fundamental in the process of evaluation of land ecological security. This is because an ecosystem is a complex system made up of natural, economic, and social components. The relationships between indicators, which can include all aspects of the economy, society, and environment, must be taken into account to reflect the land ecological security of a study area. It is important to take into account the accessibility of indicator data within this approach [39]. The present investigation selected 19 indicators based on data accessibility, dynamics, experimental science, and the index system [40,41]. Changes in the ecological environment are driven primarily by social development [42]. The population status and level of economic development are selected as driving factors for assessing ecological security. The pressure is directly caused by the driving force, which is the direct result of human activities. Population density, the amounts of fertilizer and pesticides applied, and the built-up area are the most obvious factors contributing to the alteration of the ecological environment. The state is the stage at which the ecosystem is developing under the influence of forces and pressures, and it can be represented by variables such as temperature, precipitation, cultivated land, and farmer income. The impact is the effect of changes in the ecological environment on human society, such as grain yield and the rate of green coverage in urban areas. Response is a series of measures taken when ecological security is threatened, including afforestation, industrial wastewater and solid waste discharge and treatment rate [43].The specific indicators are shown in Table 3.

**Table 3:** Indicators of ecological security and their respective weights in the study area.

Project	Index	Description	unit	Symbol (Beneficial,+; Costly,-)	Weight(Kunming)	Weight(Fuzhou)
**Driving** **(D)**	Per capita GDP	Indicates the level of socio-economic development	Yuan / person	+	0.0890	0.0723
	Urbanization rate	Indicates the degree of population aggregation to the city.	%	-	0.0494	0.0454
	Natural population growth rate	Indicates the trend of population growth	‰	-	0.0326	0.0327
	Annual GDP growth rate	Indicates the trend of economic growth	%	+	0.0682	0.0954
**Pressure** **(P)**	Population density	Indicates the pressure of population on land	Person/km^2^	-	0.0344	0.0315
	Fertilizer application rate	The pressure of agricultural production on the ecological environment	10,000 tons	-	0.0498	0.0655
	Pesticide application rate	The pressure of agricultural production on the ecological environment	10,000 tons	-	0.0434	0.0745
	Built-up area	Indicates the pressure of urban development on the ecological environment.	km^2^	-	0.0526	0.0491
**State** **(S)**	Cultivated area	Indicates the quality of regional land resources.	10,000 hectares	+	0.0449	0.0318
	Annual average precipitation	Indicates the amount of regional precipitation	mm	+	0.0333	0.0369
	Annual average temperature	Indicates the degree of air cooling and heating	°C	+	0.0525	0.0633
	Per capita net income of farmers	Indicates the economic consumption level of residents.	yuan	+	0.0924	0.0694
**Impact** **(I)**	Per capita grain production	Indicates soil quality, soil fertility and soil health.	ton/person	+	0.0331	0.0703
	Per capita park green area	Indicates the state of urban resources.	m^2^	+	0.0422	0.0407
	The proportion of the tertiary industry	Indicates regional industrial structure.	%	+	0.0507	0.0836
	Green coverage rate of urban construction area	Indicates the growth state of regional vegetation.	%	+	0.0506	0.0283
**Response** **(R)**	Afforestation area of the year	Indicates the direct response of the regions to environmental improvement	hectare	+	0.0882	0.0419
	Disposal utilization rate of industrial solid waste	An indication of regional efforts to protect the environment	%	+	0.0594	0.0291
	Ratio of industrial wastewater discharge to total wastewater discharge	Indicates that the region attaches importance to ecological security.	%	-	0.0334	0.0385

### (2) Standardization of data

As the indicators are derived from multiple levels, such as society, nature, and the economy, it is impossible to directly compare ecological security indicators. Therefore, these indicators must be standardized prior to evaluation. The standardization of positive indicators is performed as follows:

$$X_{ij}^{\prime }=\frac{{\text{x}}_{\text{ij}}-{\text{x}}_{\min}}{{\text{x}}_{\max}-{\text{x}}_{\min}},$$ (3)

Standardization of negative indicators is performed as follows:

$$X_{ij}^{\prime }=\frac{{\text{x}}_{\text{max}}-{\text{x}}_{\text{ij}}}{{\text{x}}_{\max}-{\text{x}}_{\min}},$$ (4)

where *x*_*ij*_ is the index value of item *j* in the year *i*; *x*_*max*_ and *x*_*min*_ are the maximum and minimum values of index *j*, respectively; and $X_{ij}^{\prime }$ is a standardized value.

### (3) Utilization of the entropy weight method to determine the weight of each index

This study evaluated the dispersion of indicators based on the properties of entropy to determine the weight of each indicator. The entropy value of index e_j_ was calculated as follows [44]:

$$e_{j}=k\sum_{i=1}^{n}\frac{x_{ij}^{\prime }}{\sum_{i=1}^{n}X_{ij}^{\prime }}\text{In}\left(\frac{x_{ij}^{\prime }}{\sum_{i=1}^{n}X_{ij}^{\prime }}\right)$$ (5)

The weight of the j-th index was calculated as follows:

$$w_{j}=\frac{1-e_{i}}{\sum_{i=1}^{n}1-e_{i}},$$ (6)

where *e*_*j*_ is the entropy value of the *j-th* index; *w*_*j*_ is the weight of the *j-th* index; and $X_{ij}^{\prime }$ is the standardized value. These equations were utilized to determine the weights of the indicators in the study area (Table 3).

### (4) Calculation of the comprehensive assessment index for ecological security


$$ESI=\sum_{j=1}^{n}X_{ij}^{\prime }\times w_{j},$$ (7)

where *ESI* is the ecological security index; $X_{ij}^{\prime }$ is the standardized value; *w*_*j*_ is the weight of the *j-th* index; and *n* is the number of evaluation indices.

### (5) Determination of the ecological safety level

There is no global or Chinese unified standard for evaluating the ecological security level [45]. To provide an accurate and objective reflection of the ecological security of the study area, the current study referred to both research in China and abroad [46–48]. By combining the characteristics of natural resources and the ecological environment in the study area, five levels of ecological security were defined (Table 4).

**Table 4:** Classification criterion for the evaluation of the ecological security index grade.

Ecological security index value	Ecological safety level	Ecological security status	Ecosystem characteristics
(0, 0.3]	V	very unsafe	The structure of the ecological environment is incomplete, there are many ecological disasters, and it is difficult to restore the ecological environment
(0.3, 0.5)	IV	Unsafe	The ecological environment is destroyed, but the basic functions are sound, and there are ecological disasters from time to time
[0.5, 0.6]	III	general safety	The ecological environment is in general, the concept of environmental protection is strengthened, and the ecological environment is being improved
(0.6, 0.8]	II	relatively safe	The ecological environment is in good condition, and the ecosystem can be restored under normal disturbance
(0.8, 1]	I	very safe	The ecological environment is in good condition, the ecosystem is highly functional, and the ecosystem has a strong ability to restore

#### 2.3.4 Method of gray relational analysis

The gray relational analysis is a subfield of gray systems theory that can be applied to determine the degree of correlation between an indicator and other indicators in a system where only a portion of the information is clear [49]. Following are the steps of the method: (1) selecting the data for dimensionless processing; (2) establishing the reference sequence and comparison sequence; and (3) calculating the correlation coefficient and correlation degree.

The correlation coefficient is calculated as follows:

$${\text{S}}_{\text{ok}}(\text{t})=\frac{\Delta \min+\rho \Delta \max}{\Delta_{\text{ok}}(\text{t})+\rho \Delta \max},$$ (8)

where *S_0k_(t)* is the correlation between factor *x*_*k*_ to factor *x*_*o*_ at time t and *ρ* is the gray number on the interval [0,1], which is assumed to be 0.5 for this study. The degree of correlation is calculated as follows:

$$\gamma_{\text{ok}}=\frac{1}{\text{m}}\sum_{\text{i=1}}^{\text{m}}\;{\text{S}}_{\text{ok}}(\text{t}),$$ (9)

where γ_ok_ is the correlation between x_*k*_ and *x*_*o*_. The correlation has a positive relationship with the evaluation object’s significance in relation to the evaluation criteria.

## 3. Results

### 3.1 Comparative analysis of land use classification results

**Fig 2:**
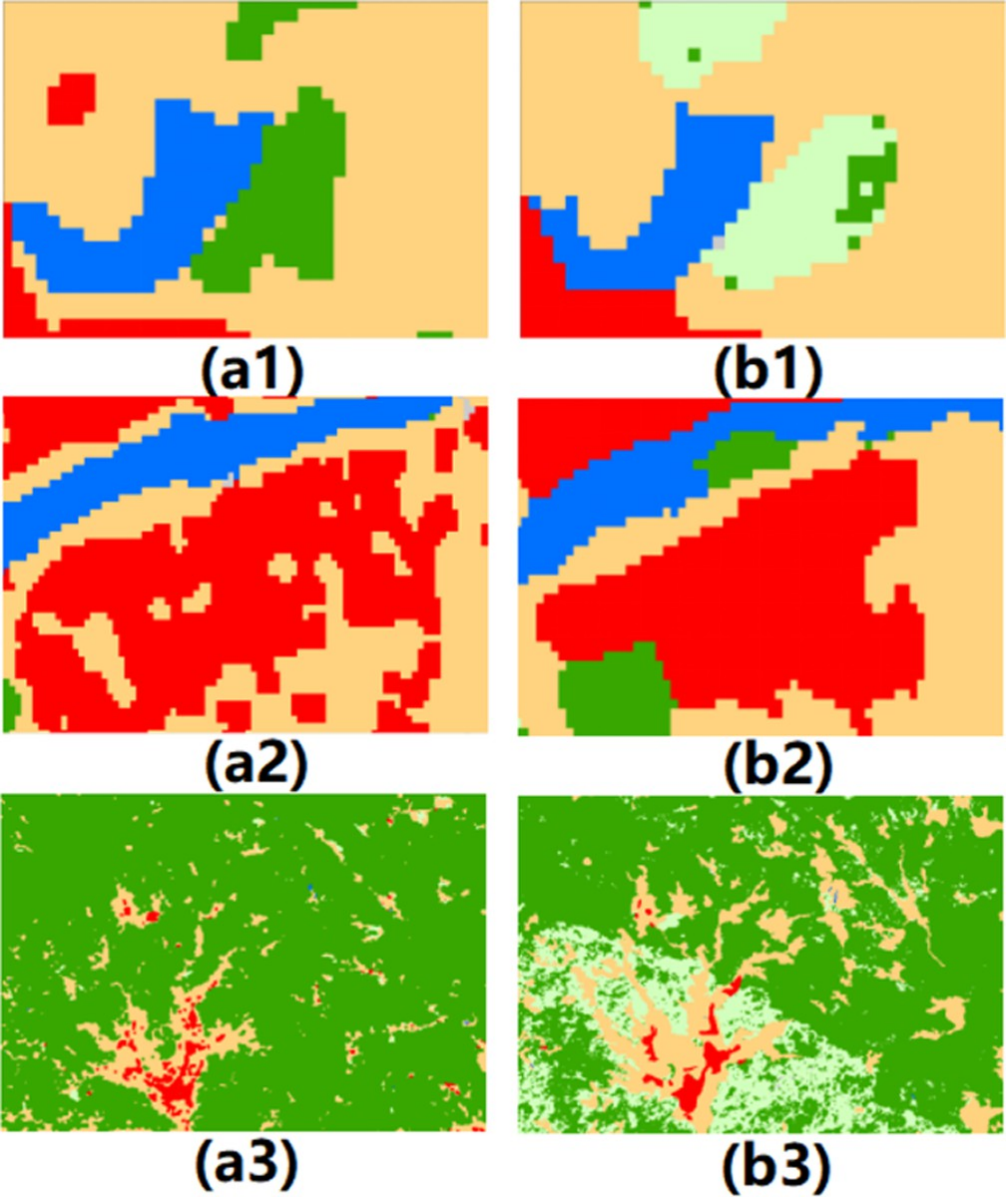
Detailed classification results of different land cover classification products. a shows the classification result of the present study; b shows the Globe Land30 data after reclassification; 1, 2, and 3 represent three different geographical positions.

The high-precision RF classification analysis results were utilized in the current study (Table 2). The results of the present study’s classification (Fig 2A) was checked against Globe Land30 data (Fig 2B) developed by the National Basic Geographic Information Center [50]. The Globe Land30 data had previously been reclassified to be consistent with the results of the present study. The findings demonstrated that the classification produced by the present study was consistent with the Globe Land30 in terms of both the spatial distributions of different types of land features and the areas and proportions of each land cover type.However, by selecting multiple small areas for visual analysis, it is found that the classification achieved in the present study was more accurate than that of the Globe Land30 for regions exhibiting a relatively fragmented distribution of land use types. This result confirmed that the classification performed in the current study could provide more accurate data support for future analyses (Fig 2).

### 3.2 LUCC in east and west subtropical China

**Fig 3:**
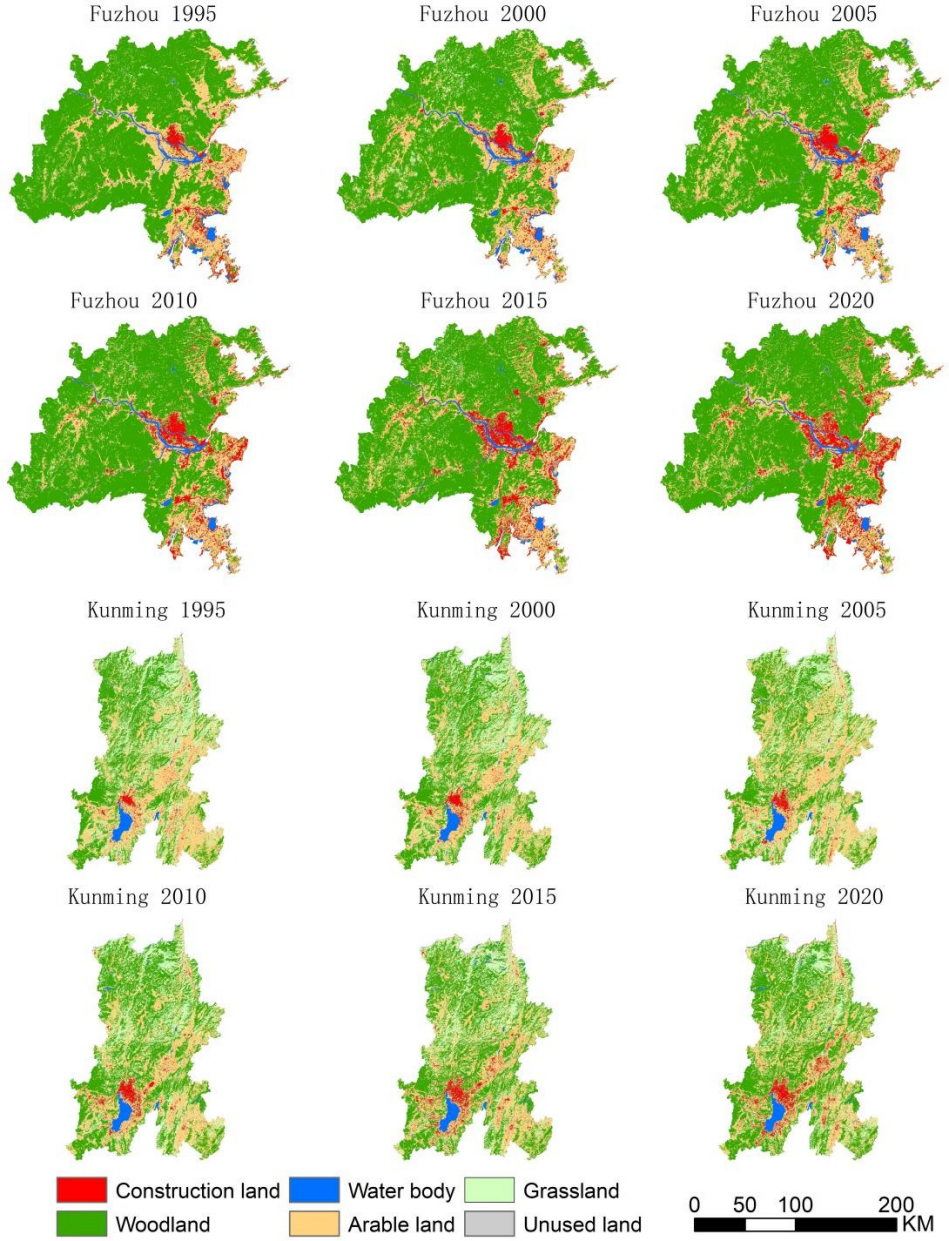
Land use classification map of Kunming and Fuzhou.

The distributions and areas of land use types in the study area in 1995, 2000, 2005, 2010, 2015, and 2020 are depicted in Fig 3 and Table 5. The results indicated that woodland and arable land were the predominant types of land use in the two cities, with unused land comprising less than 1% of the total area of each city. The majority of the construction land was situated near water bodies like lakes and rivers. Although Kunming had a larger grassland area than Fuzhou, Fuzhou had a greater quantity of forest resources that were also more widely distributed. Lakes were the predominant source of water in Kunming, whereas rivers dominated in Fuzhou.

**Table 5:** Areas of different land use types in Kunming and Fuzhou in km^2^.

City	Land use/land cover type	1995	2000	2005	2010	2015	2020
**Kunming**	**Construction land**	603.60	653.82	661.04	789.86	1097.53	1137.11
	**Woodland**	7476.59	8342.61	7129.93	8525.96	8178.83	9361.07
	**Water body**	444.42	417.25	405.31	483.21	519.07	497.58
	**Arable land**	7227.79	6490.87	7995.23	6536.98	6599.16	6245.45
	**Grassland**	5172.43	5018.74	4763.97	4668.74	4524.33	3722.10
	**Unused land**	98.03	99.57	67.38	18.11	103.94	59.55
**Fuzhou**	**Construction land**	543.03	579.60	735.46	883.92	969.98	1110.26
	**Woodland**	7347.99	7080.69	7081.14	7351.95	7131.46	7291.31
	**Water body**	316.20	356.79	337.85	311.44	281.68	252.61
	**Arable land**	2653.44	2583.84	2588.49	2252.36	2402.95	2231.33
	**Grassland**	231.73	536.36	377.37	341.39	342.21	232.83
	**Unused land**	57.28	12.41	29.37	8.62	21.41	31.34

**Fig 4:**
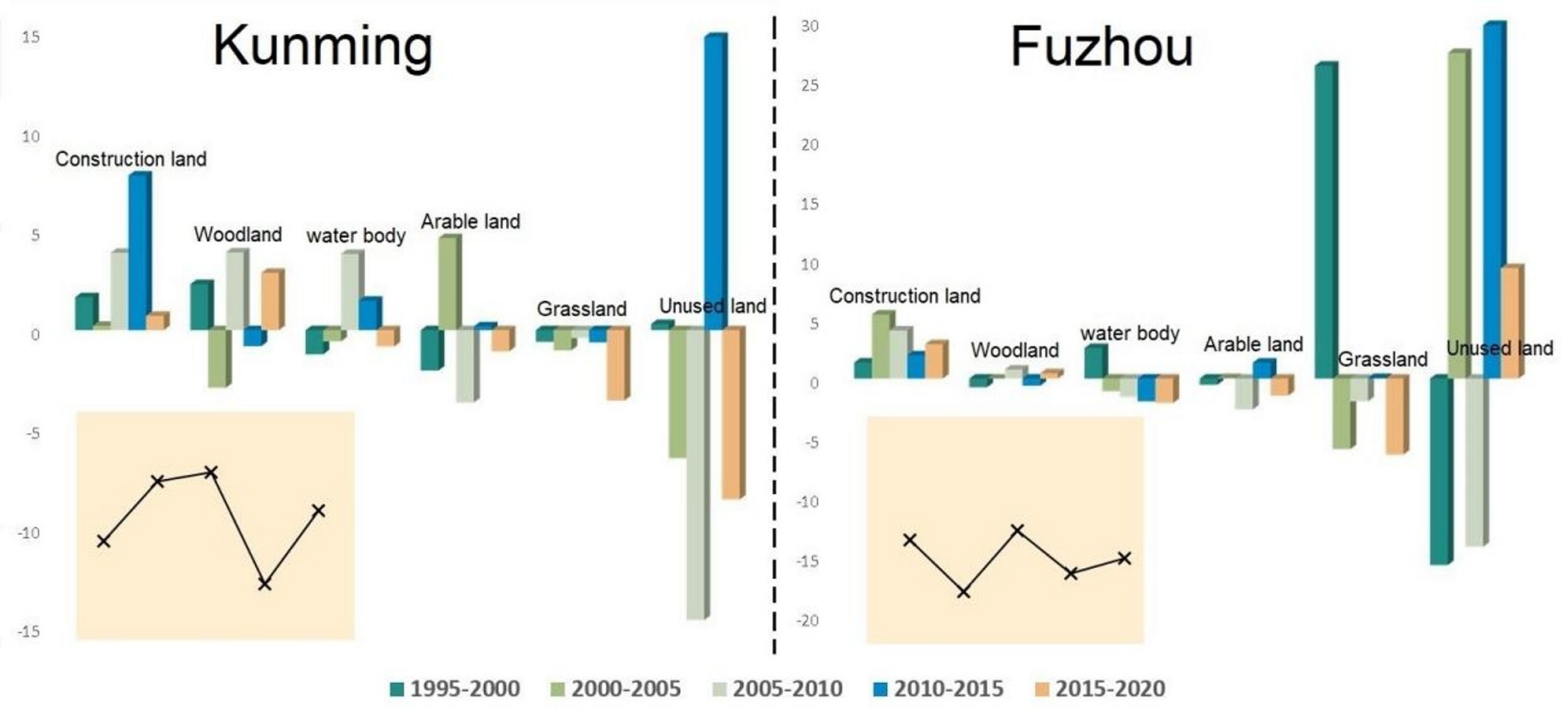
The dynamic degree of land use in Kunming and Fuzhou. The histogram shows the single dynamic degree, whereas the line graph shows the comprehensive dynamic degree.

From 1995 to 2020, Kunming’s comprehensive dynamic level of land use was higher than Fuzhou’s, with the former exhibiting more frequent changes between various land use types and more significant changes in land area and quantity (Fig 4). The study area displayed a positive dynamic degree of construction land, indicating the continuous expansion of construction land, with the index first increasing and then decreasing. However, the expansion of construction land began to increase sooner in Fuzhou City than in Kunming City. The dynamic degree of woodland in Kunming clearly exceeded that of Fuzhou, indicating that the woodland resources in Fuzhou were comparatively stable. The dynamic degree of water bodies in Fuzhou was higher than that in Kunming, and the former city displaying a clear downward trend in the area covered by water bodies. The dynamics of arable land exhibited a declining trend, and arable land continued to decrease in both cities. Although the dynamic degree of unused land and grassland fluctuated considerably, these two land use types comprised only a small proportion of the total land area, resulting in a negligible impact on LUCC.

### 3.3 Spatiotemporal pattern of ecological security

**Fig 5:**
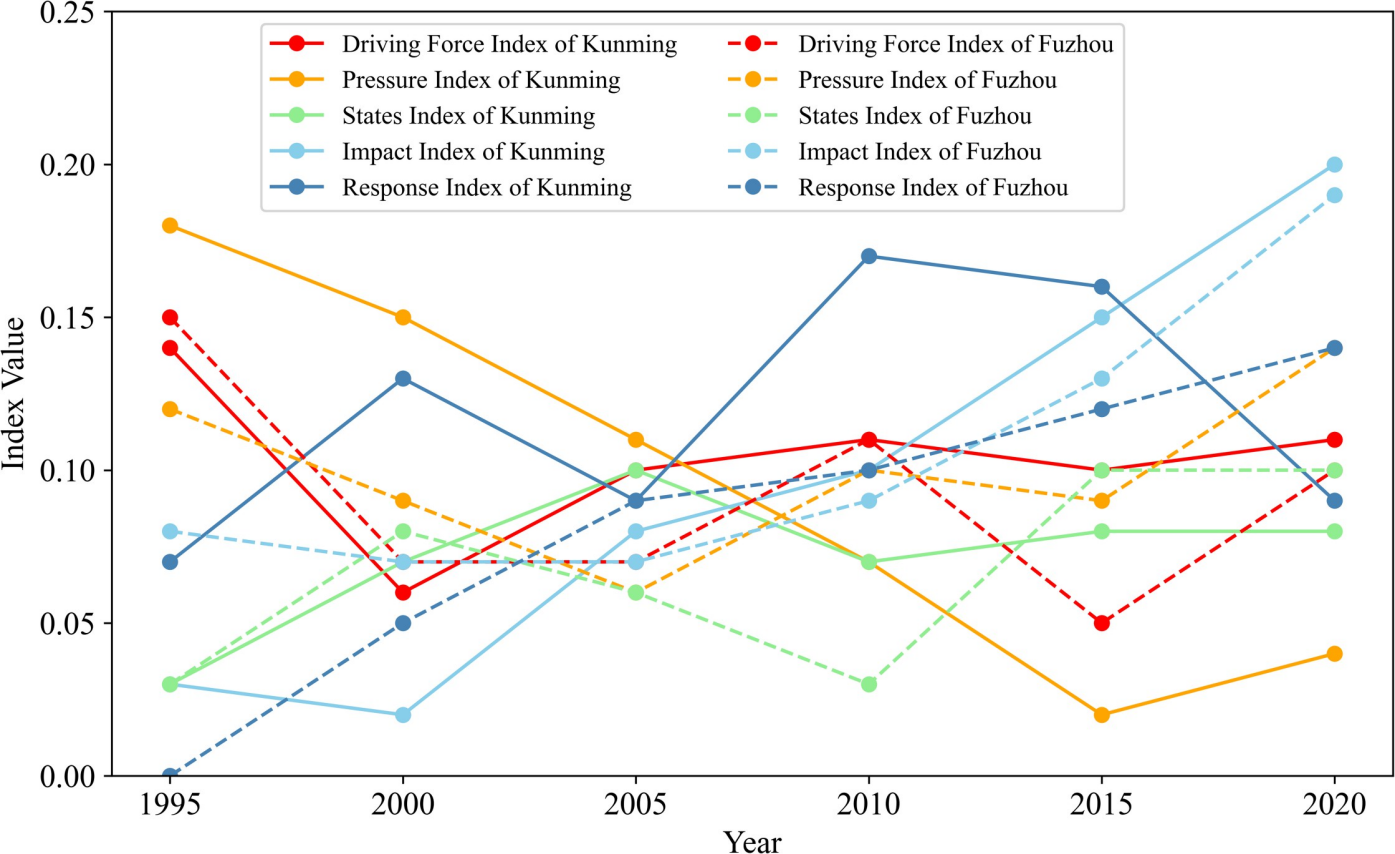
Changes in ecological security index values of subsystems in Kunming and Fuzhou.

The ecological security index values for Kunming and Fuzhou were calculated using the selected indicators, as displayed in Table 6. From 1995 to 2020, the ecological security index values of the two cities exhibited overall upward trends. Although there have been gradual improvements in the ecological security of the two cities, there is still room for further development. The ecological security index of Kunming increased from 0.42 to 0.52, signifying a change in ecological security status from unsafe to generally safe. From 0.36 (unsafe), Fuzhou City’s ecological security index increased to 0.68 (relatively safe). The level of ecological security has steadily increased, and the state of ecological security as a whole improved. As illustrated in Fig 5, the impact index increased in both cities, while the pressure index and response index increased in Fuzhou City, the driving force index decreased in Kunming City, and there were variations in the ecological security indices of other subsystems.

**Table 6:** Ecological security comprehensive index and safety level each year.

	1995	2000	2005	2010	2015	2020
**Kunming**	0.45	0.42	0.48	0.51	0.52	0.51
	unsafe(IV)	unsafe(IV)	unsafe(IV)	generally safe(III)	generally safe(III)	generally safe(III)
**Fuzhou**	0.38	0.36	0.36	0.43	0.50	0.68
	unsafe(IV)	unsafe(IV)	unsafe(IV)	unsafe(IV)	generally safe(III)	relatively safe(II)

### 3.4 Correlation between LUCC and ecological security

The average ecological security index value for two consecutive years was used as the reference sequence, while the single dynamic degree of each land use type was used as the comparison sequence. These data were then processed in a dimensionless manner using the mean value method, and the degree of correlation between the ecological security index and the single dynamic degree of land use was calculated using Eq (9). (Table 7). In both cities, the correlation between construction land dynamics and the ecological security index was greater than 0.9, indicating a strong relationship between the two variables. Kunming had the strongest and third-strongest relationships between the ecological security index and grassland and woodland dynamics, while Fuzhou had the fifth and sixth strongest, respectively. The area and distribution of woodlands and grasslands in Fuzhou changed little during the study period. Therefore, the relationships between these two types of land cover and the ecological security index were weak. However, there were strong correlations between the ecological security index and the consistently negative dynamic degree of grassland, and the fluctuating dynamic degree of forest land in Kunming. The proportion of unused land area was less than 1%, and the high dynamic degree of this land use type could be attributed to fragmentation-related difficulties in classifying remote-sensing images. However, this land use type had a negligible effect on ecological security. In comparison to Kunming City, the relationships between the ecological security index and the dynamic levels of water bodies and arable land in Fuzhou City were stronger.

**Table 7:** Correlation degree between the ecological security index and the single dynamic degree for different land uses.

	Correlation(Kunming)	Rank	Correlation(Fuzhou)	Rank
**Construction land**	0.909	2	0.971	1
**Woodland**	0.768	3	0.499	6
**Water bodies**	0.704	5	0.915	2
**Arable land**	0.602	6	0.915	3
**Grassland**	0.920	1	0.822	5
**Unused land**	0.763	4	0.878	4

The present study utilized the ecological security index values of the two cities as a reference sequence and the land cover type areas as a comparison sequence. The correlations between the ecological security index and the areas of land cover types were then calculated using the same method (Table 8). Water bodies and woodland showed significantly higher correlations with the ecological security index in Kunming, followed by arable land, grassland, and construction land, with unused land demonstrating the lowest correlation. Although water bodies comprised only 2.2% of the total land area of Kunming, this land use type was closely associated with ecological security. The areas of arable land and grassland showed similar correlations with the ecological security index, and the impact on ecological security is in the middle level. The correlation between woodland and the ecological security index reached 0.87, which could be attributed to the large area of woodland (39%). According to their correlations with the ecological security index, the different land use types in Fuzhou were ranked as follows: construction land > woodland > arable land > water body > grassland > unused land. From 1995 to 2020, the construction land area in Fuzhou nearly doubled, which had a significant impact on the city’s ecological security. The proportion of land covered by woodland in Fuzhou remained relatively constant throughout the study, accounting for an average of 65% of the city’s total land area. Therefore, woodland played a significant role in maintaining the ecosystem’s stability. In contrast, grassland and unused land comprised a small portion of the total land area. Consequently, they had minimal effects on ecological security.

**Table 8:** Correlation degree between the ecological security index and the areas of various land cover types.

	Correlation degree(Kunming)	Rank	Correlation degree(Fuzhou)	Rank
**Construction land**	0.713	5	0.887	1
**Woodland**	0.870	2	0.817	2
**Water bodies**	0.934	1	0.752	4
**Arable land**	0.792	3	0.784	3
**Grassland**	0.784	4	0.731	5
**Unused land**	0.566	6	0.618	6

The results presented in Tables 7 and 8 indicate that woodlands comprised the greatest proportion of land area in both cities and that this land use type exhibited a strong correlation with the ecological security index. The dynamic degree of woodland in Fuzhou was less than 1%, and there was little correlation between woodland and the ecological security index. However, the dynamics of woodlands in Kunming fluctuated significantly, resulting in a strong correlation between woodlands and the ecological security index. The ecological security index had the strongest correlation with the area of construction land in Fuzhou. In the past 25 years, the amount of construction land in Fuzhou has expanded rapidly, resulting in a major impact on the urban ecological security level. Although water bodies comprised less than 3% of the total area in Kunming, this land cover type demonstrated a strong correlation with the ecological security index, reaching 0.934. The improvement in the level of urban ecological security could be attributed to the comprehensive effect of LUCC. The strengths of relationships of the areas and dynamic degrees of arable land, grassland, and unused land with the ecological security index were not as high as for other features. The results indicated that any land use type occupying a large proportion of the total area or showing a large dynamic degree will have a strong correlation with the ecological security index.

## 4.Discussion

### 4.1 General overview of LUCC and ecological security in Kunming and Fuzhou

There have been several recent studies on land use change, its driving factors, and the relationship between land use change and ecological security in both Kunming [51,52] and Fuzhou [53–55]. The results show that a significant increase and decrease in construction land and arable land, respectively, in the study area, whereas the patterns of change for water bodies and unused land were unclear. In Kunming, the area of woodland and grassland increased and decreased, respectively, while woodland in Fuzhou remained relatively stable. Various studies on the health of the ecosystems in Kunming and Fuzhou have yielded varying due to the use of distinct evaluation methods and evaluation indicators. However, according to the results, the ecological security index values of both cities increased. The results of this study indicate that from 1995 to 2020, Kunming and Fuzhou experienced dramatic changes in land use, with unused land undergoing the greatest transformation, followed by an increase in construction land and a decrease in arable land, respectively. Construction land in Fuzhou and Kunming increased most rapidly in 2005–2010 and 2010–2015, respectively. The minimum ecological security index values of Kunming and Fuzhou both occurred in 2000 with values of 0.42 and 0.36, respectively, after which they increased continuously. The ecological security levels of both cities continued to improve during the study period. This indicates that the results of the present study are consistent with those of other researchers and are thus feasible.

### 4.2 Similarities and differences of ecological security responses to LUCC between the different regions

In the current study, ecological security responses to LUCC in China’s western subtropical city of Kunming and the eastern subtropical city of Fuzhou were compared and analyzed. It is found that for the coastal city of Fuzhou, the strongest correlations between the single dynamic degree of land use and the ecological security index, and the correlation between the area of each land cover type and the ecological security index, are all construction land. For the inland city of Kunming, the strongest correlation is the water body. The correlation between the woodland area and the ecological security index is strong in both places. Normal ecosystem service functions are guaranteed in a healthy forest ecosystem, and reasonable human development is supported by this type of ecosystem. Within the entire urban ecosystem, forest ecosystems also contribute to the regulation of climate, the preservation of water and soil, and the sequestration of carbon [56]. In both Kunming and Fuzhou, woodland areas account for the majority of the land. In recent years, Kunming and Fuzhou have implemented a policy of "returning farmland to forests," which has involved the widespread planting of trees. Large-scale afforestation may be the main reason for the improvement of urban ecological security. Due to its convenient transportation, pleasant climate and other regional advantages, Fuzhou has witnessed rapid economic development and continuous expansion of construction land. Relevant scholars [57,58] have studied the impact of impervious surface on ecological security in Fuzhou in recent years, and found that the increase of impervious surface has a negative impact on urban ecological security to a certain extent. In the past 25 years, the annual precipitation in Kunming has fluctuated greatly, and the population of the main urban area has grown rapidly. Environmental issues like water shortages and severe pollution have become more prominent as a result of the area’s promotion of development around lakes. Research [59] has shown that the deterioration of water ecological environment has affected the improvement of the overall ecological environment in Kunming city.

### 4.3 Limitations and future work

LUCC has a close relationship with ecological security. The development and utilization of land differ between regions. As a result, different regions will have different ecological security responses to LUCC. In the current study, ecological security responses to LUCC for two provincial capital cities in China’s east and west subtropical zones were analyzed and compared on a macro-level. The findings can be used to direct similar comparative studies across more regions. However, due to a lack of data, an analysis of the ecological security of the districts and counties that were included in the study could not be conducted. The different districts and counties in the study area have very different natural environments, levels of economic development, and social environments. The differences in land development and utilization among the different districts and counties can result in a lack of inconsistency in the levels of ecological security within the region. For example, Nong and Wang (2020) [60] using the RSEI model demonstrated that the quality of the ecological environment in the west of Kunming City was superior to that in the east, with the southwest corner exhibiting the highest quality. Future research should incorporate additional evaluation methods to compensate for the lack of data, thereby enabling the evaluation of ecological security within a region. This strategy would deliver more reliable evaluation results and would highlight the variations in ecological security between various regions.

## 5.Conclusions

The present study classified land cover in Kunming and Fuzhou in 1995, 2000, 2005, 2010, 2015, and 2020 using random forest methods with the support of the GEE platform. Subsequently, 19 evaluation indices were constructed based on the DPSIR model. The ecological security of the study area was assessed and analyzed using the index sum method. Based on the findings of the present investigation, it was determined that (1) the primary land use types in Kunming and Fuzhou are woodland and arable land. Kunming and Fuzhou had average comprehensive land use dynamic degree values of 1.09% and 0.55%, respectively, from 1995 to 2020. In comparison to Fuzhou, Kunming experienced a greater change in land use. Kunming had the highest single dynamic degree for construction land, while Fuzhou had the highest single dynamic degree for grassland. Both cities had relatively low single dynamic degree values for woodland and water bodies. (2) From 1995 to 2020, the comprehensive ecological security index values for Kunming and Fuzhou increased from an unsafe level to a level of general security, indicating a substantial improvement in the ecological environment. The overall ecological security index of Fuzhou was higher than that of Kunming, indicating that the ecological environment of Fuzhou is slightly healthier than that of Kunming. The ecological security evaluation index system revealed that Fuzhou had the greatest weight of the influence layer (0.26), while Kunming had the greatest weight of the driving force layer (0.24). This result indicates that the impact index plays a significant role in evaluating the land ecological security in Fuzhou, while in Kunming, the most significant was the driving force index. (3) There is a strong correlation between LUCC and ecological security, and the correlation between the woodland and the ecological security index is very strong in both places. The expansion of construction land may be an important reason to restrain the improvement of the ecological security level in Fuzhou City, and the water resources have a great impact on the ecological security level of Kunming City.

## Supporting information

S1 File Land use cover datasets of Kunming and Fuzhou. (ZIP)
