# Supplementary figures and images for: The response of ecological security to land use change in east and west subtropical China
Source: PLoS One. 2023 Nov 16;18(11):e0294462. doi: 10.1371/journal.pone.0294462 (PMC10653418; doi:10.1371/journal.pone.0294462)

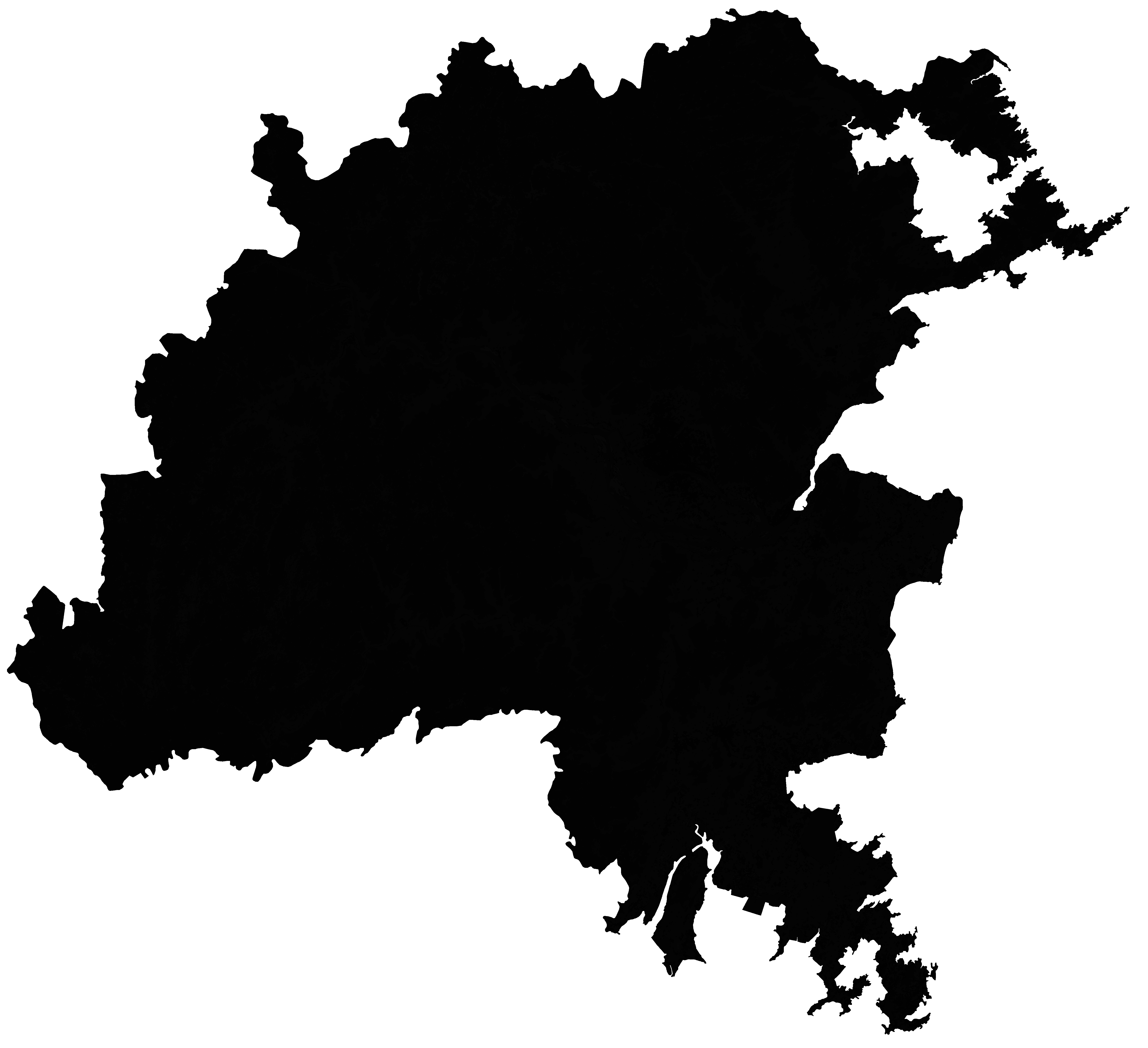

Supplement: S1 File — (ZIP) [file pone.0294462.s001.zip › S1-dataset/LUC-FZ-1995.tif]

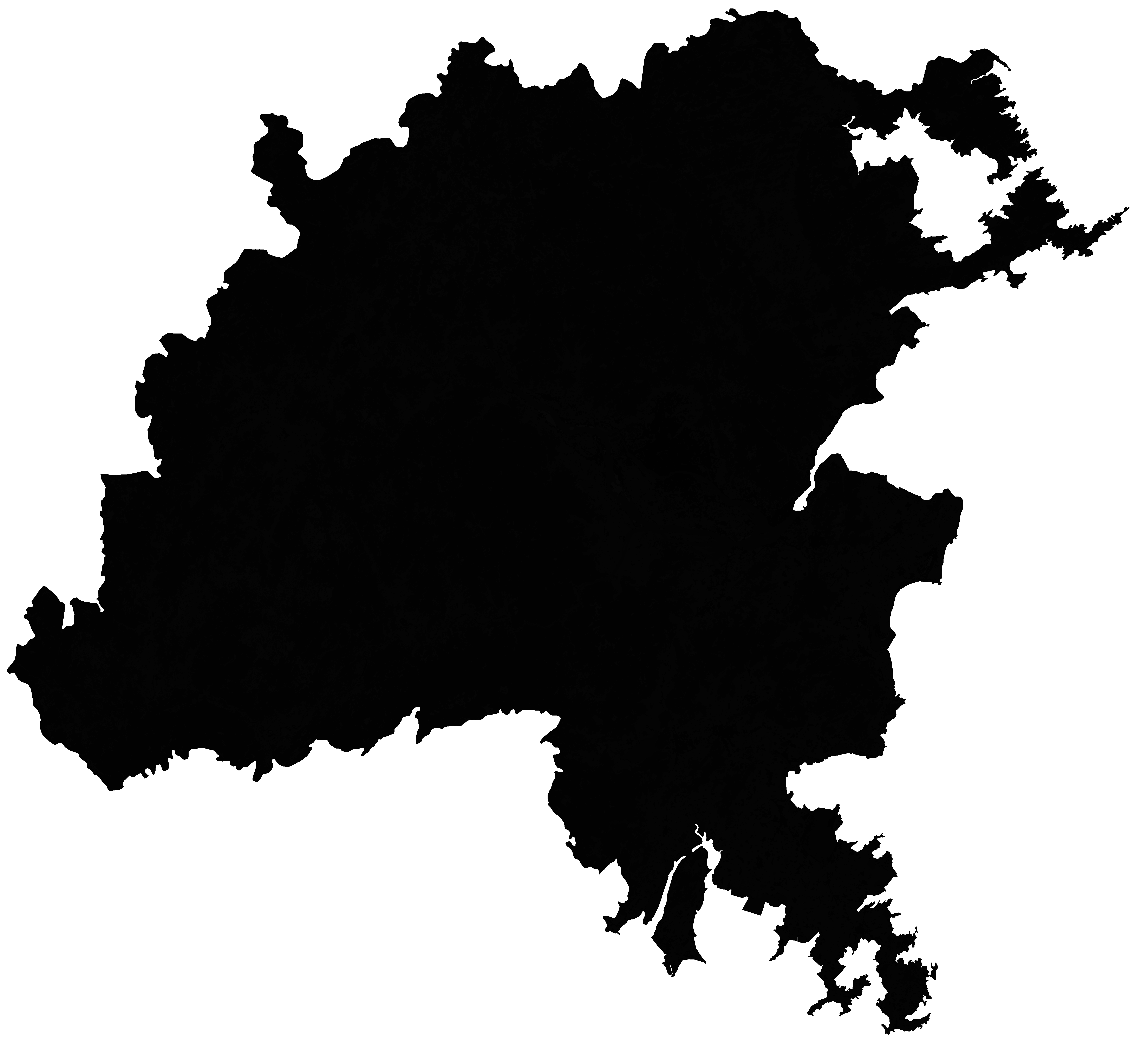

Supplement: S1 File — (ZIP) [file pone.0294462.s001.zip › S1-dataset/LUC-FZ-2000.tif]

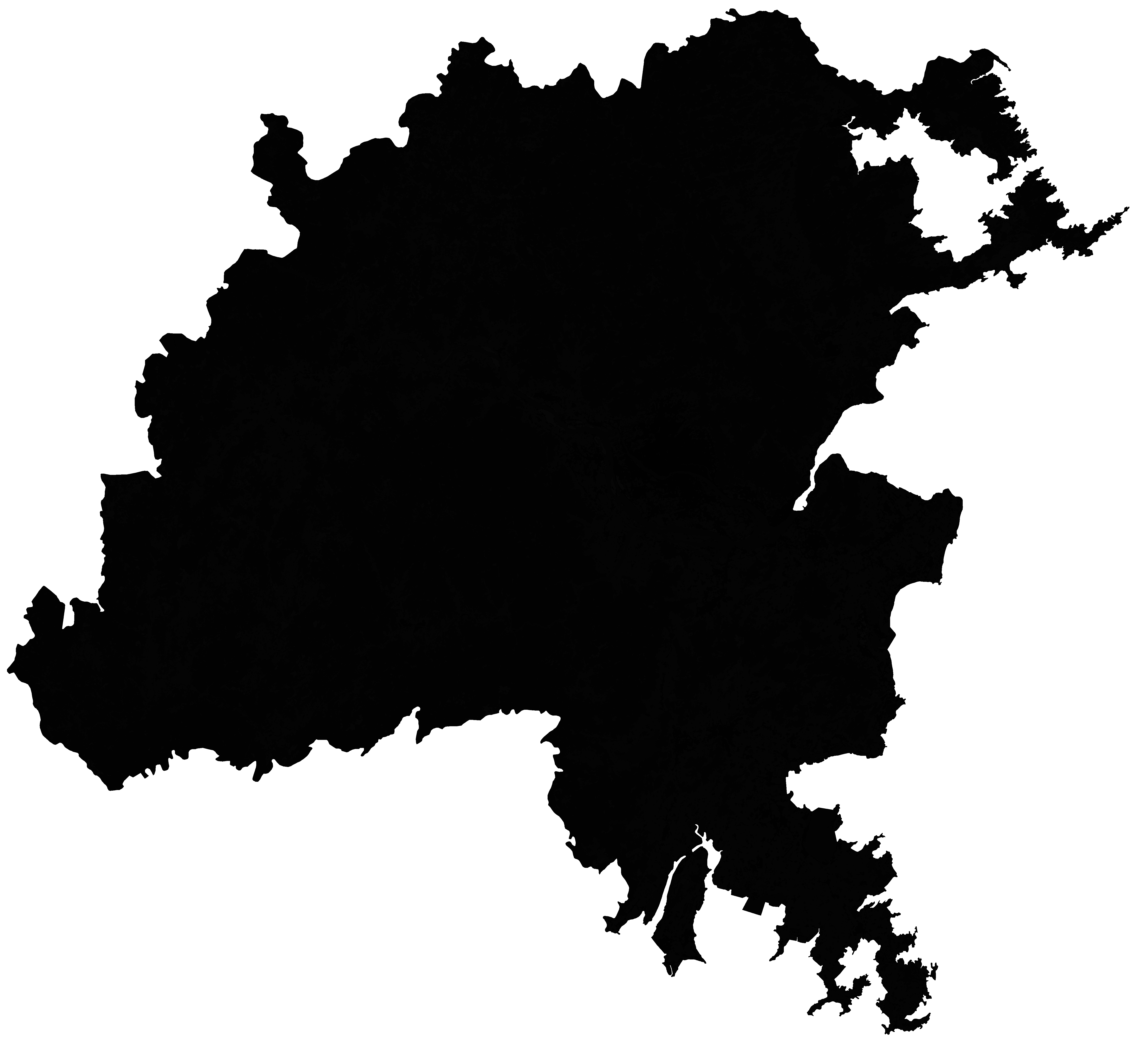

Supplement: S1 File — (ZIP) [file pone.0294462.s001.zip › S1-dataset/LUC-FZ-2005.tif]

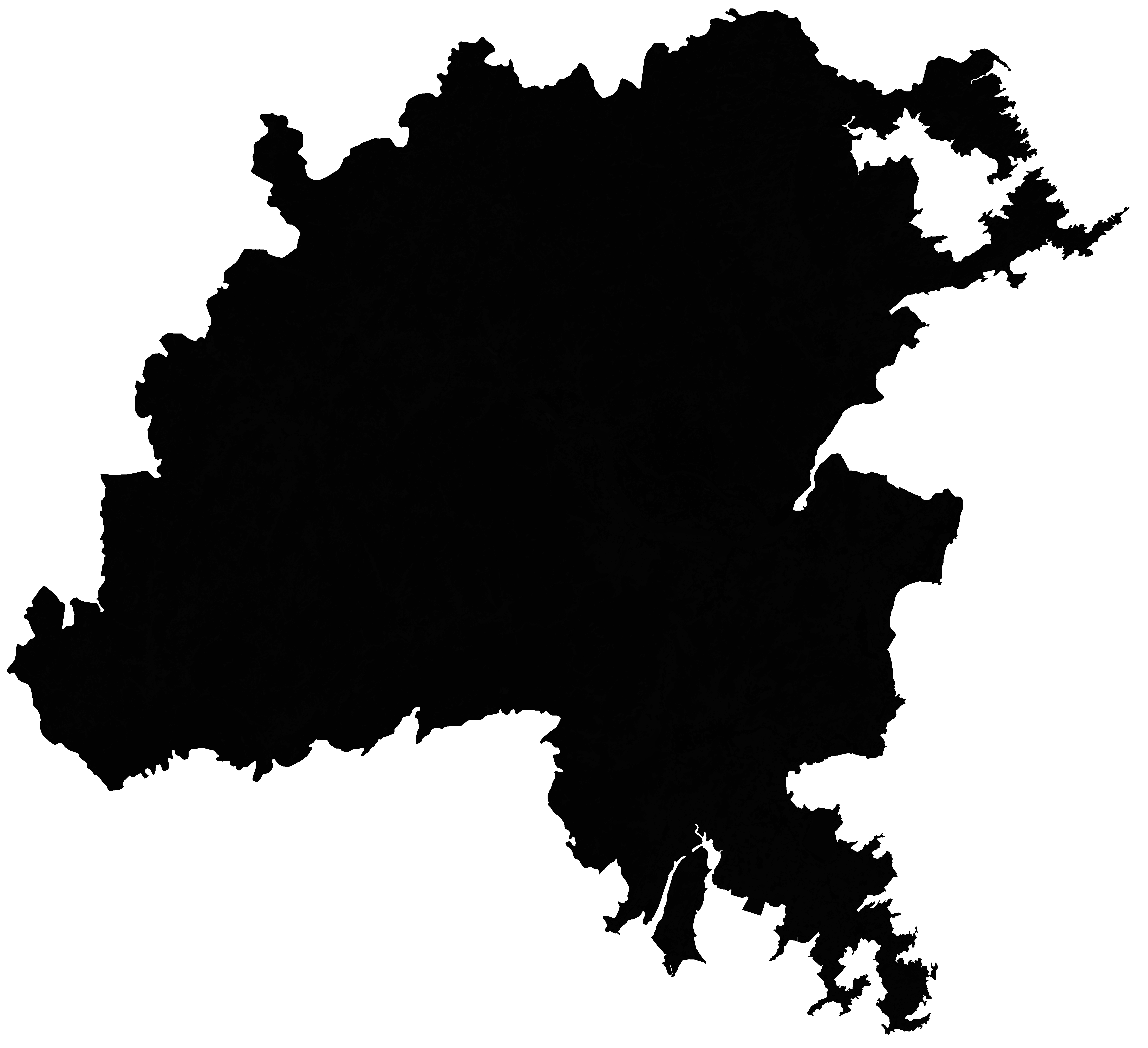

Supplement: S1 File — (ZIP) [file pone.0294462.s001.zip › S1-dataset/LUC-FZ-2010.tif]

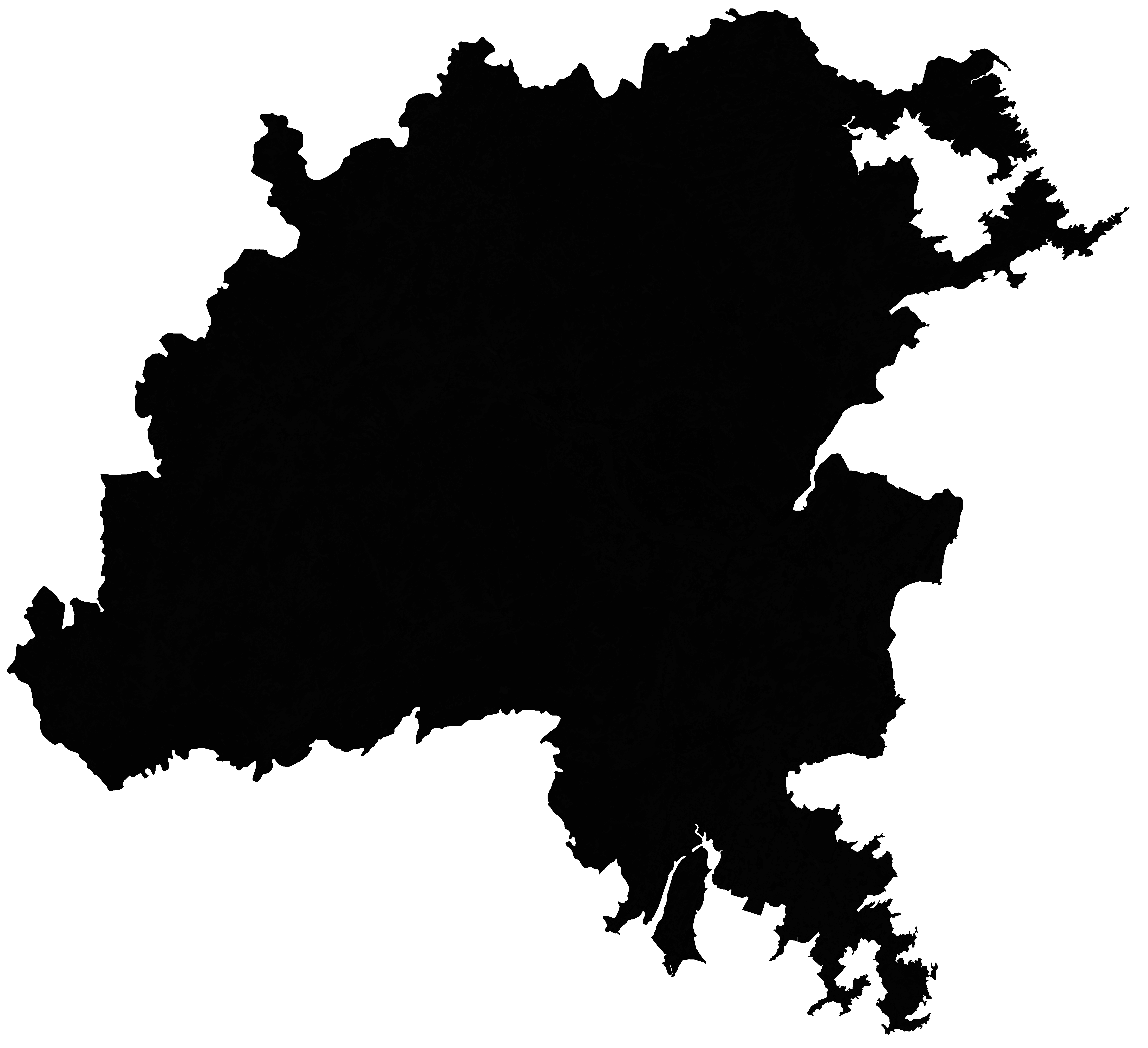

Supplement: S1 File — (ZIP) [file pone.0294462.s001.zip › S1-dataset/LUC-FZ-2015.tif]

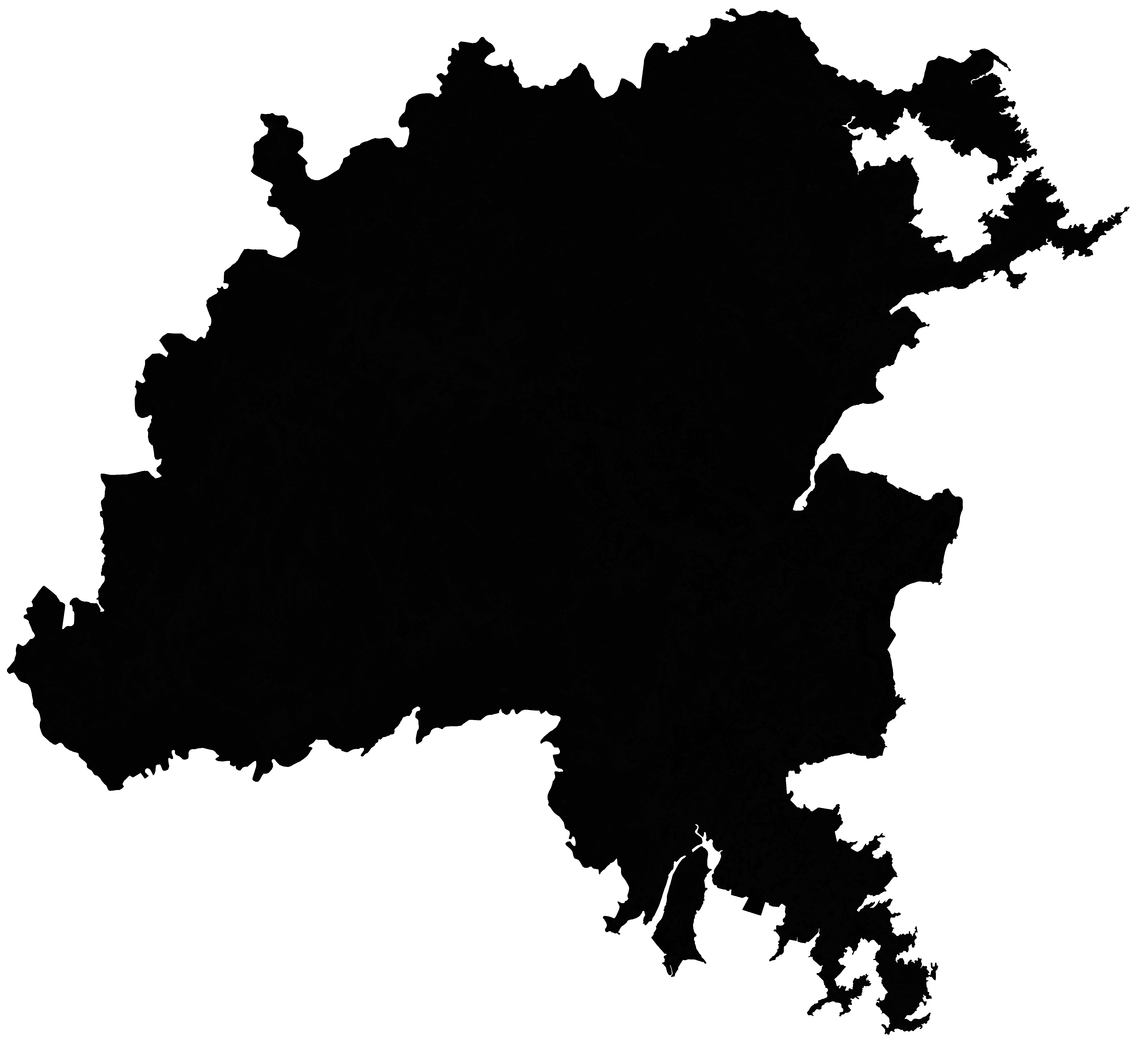

Supplement: S1 File — (ZIP) [file pone.0294462.s001.zip › S1-dataset/LUC-FZ-2020.tif]

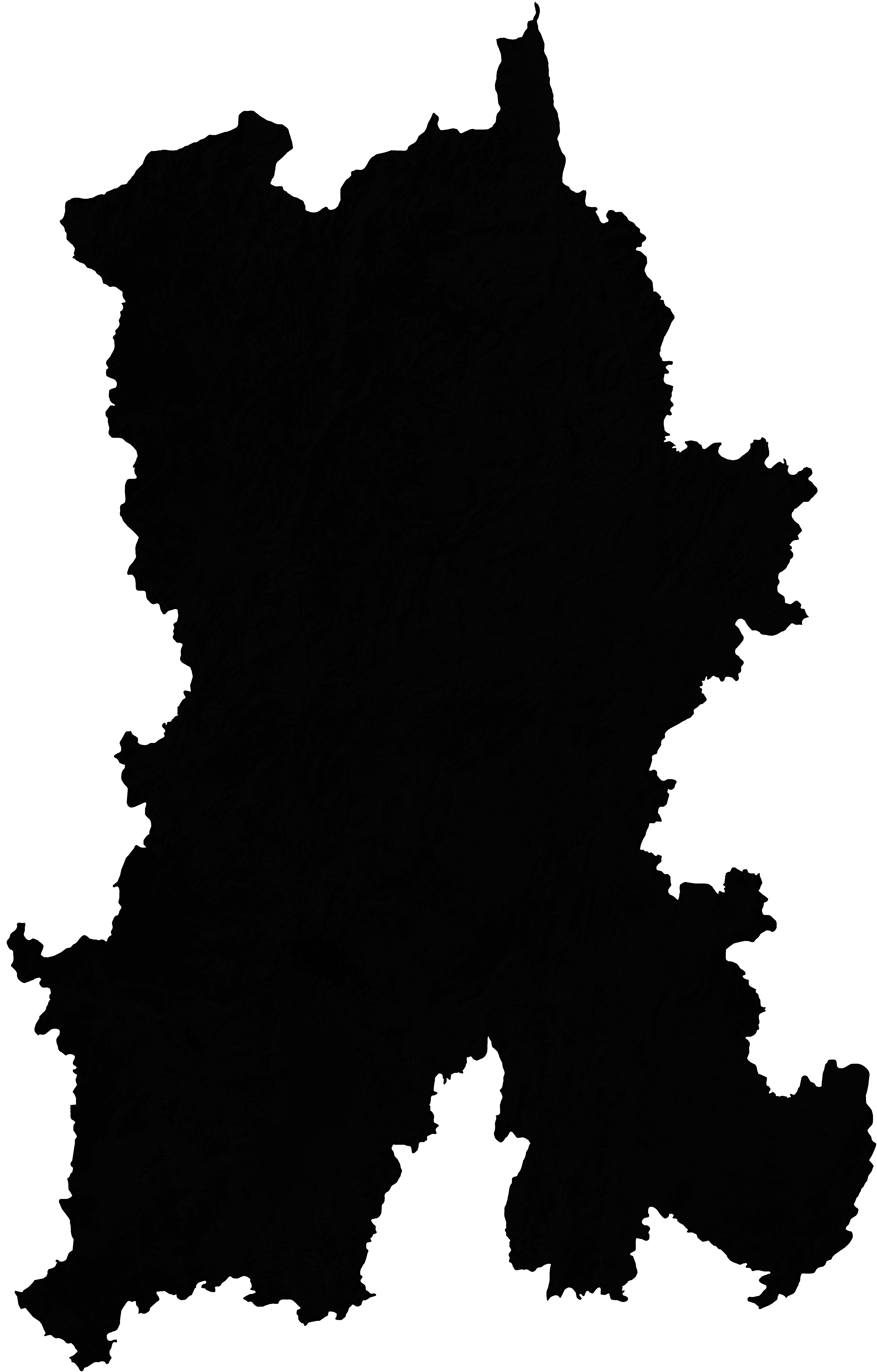

Supplement: S1 File — (ZIP) [file pone.0294462.s001.zip › S1-dataset/LUC-KM-1995.tif]

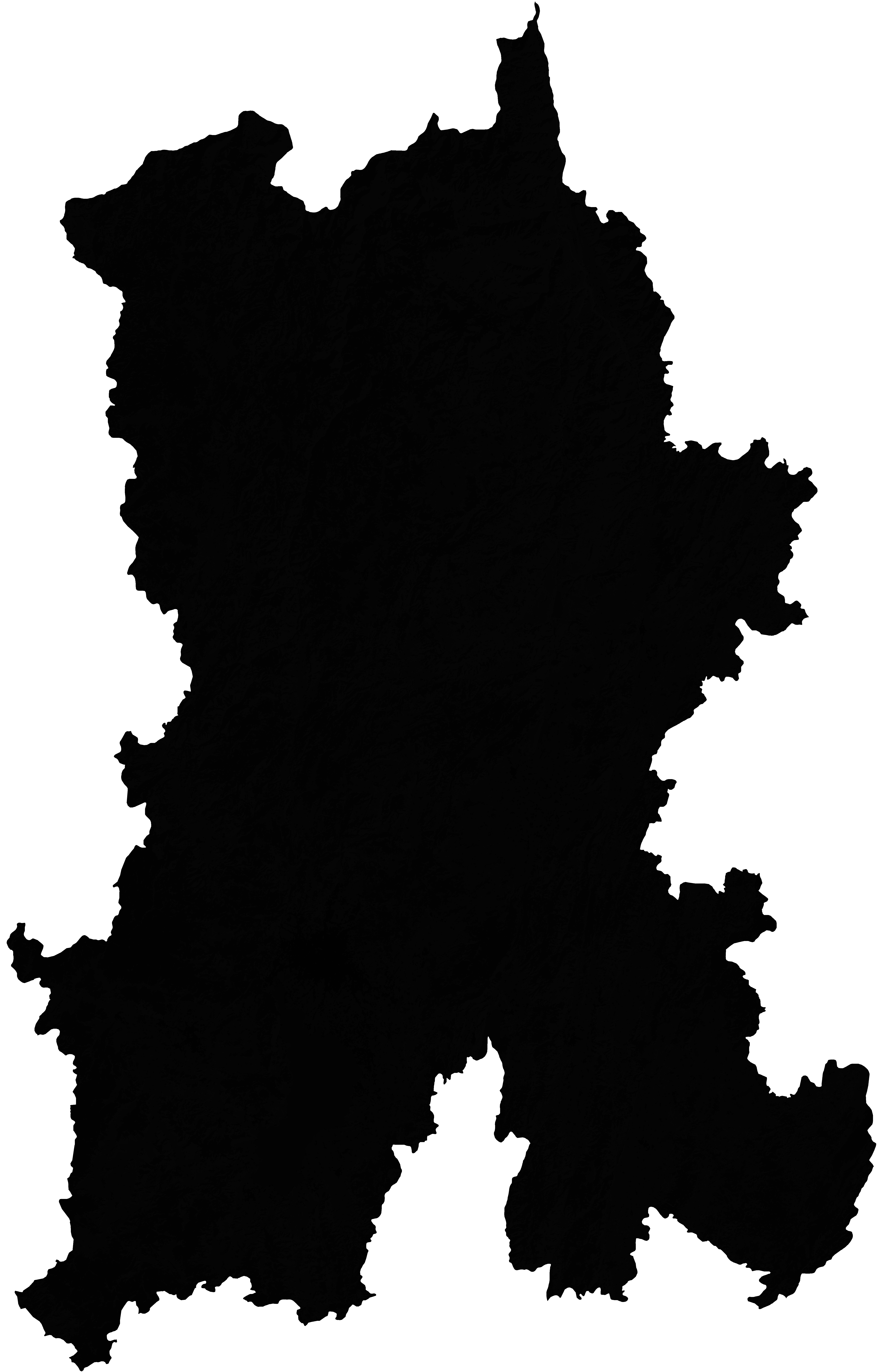

Supplement: S1 File — (ZIP) [file pone.0294462.s001.zip › S1-dataset/LUC-KM-2000.tif]

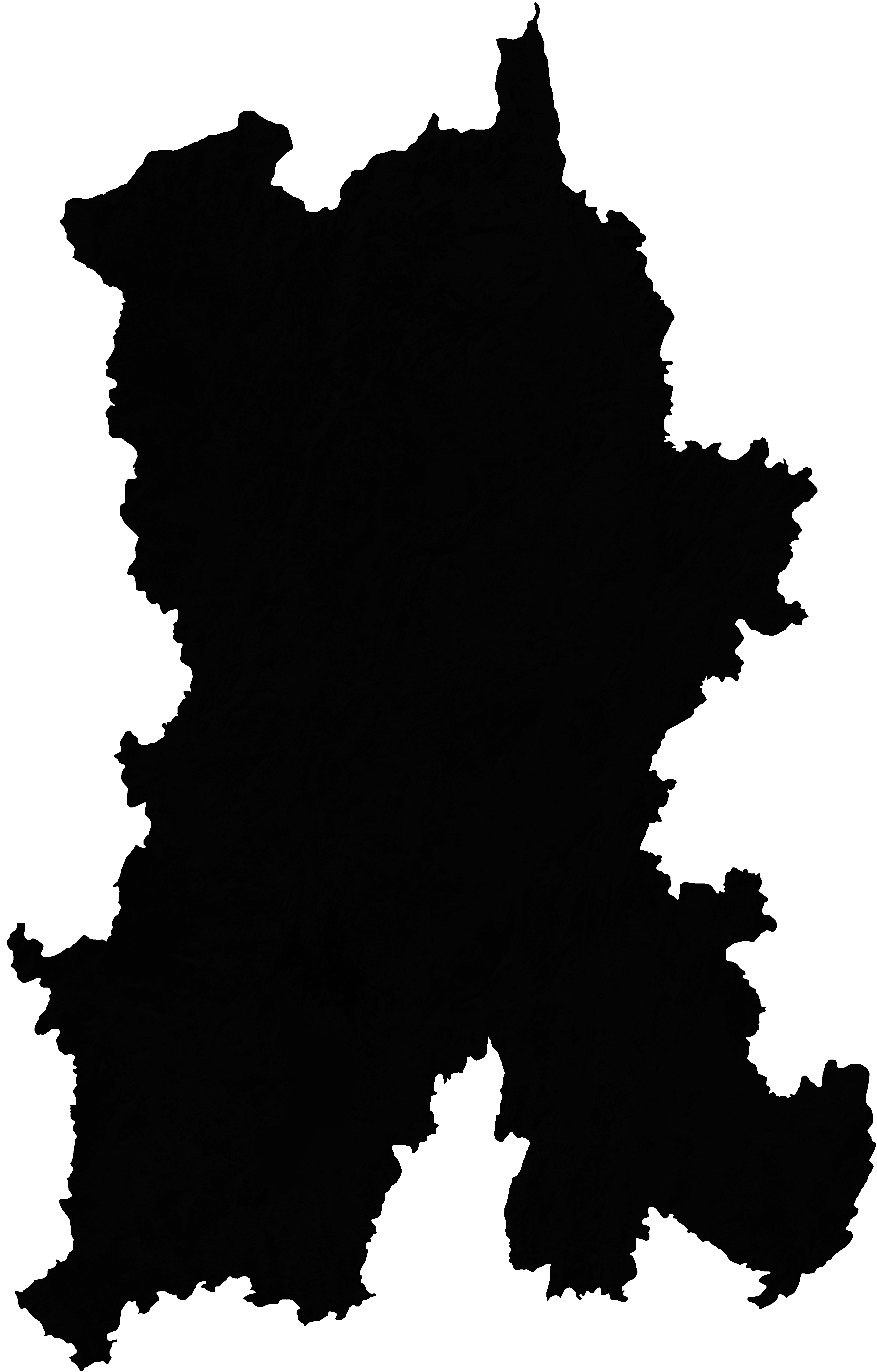

Supplement: S1 File — (ZIP) [file pone.0294462.s001.zip › S1-dataset/LUC-KM-2005.tif]

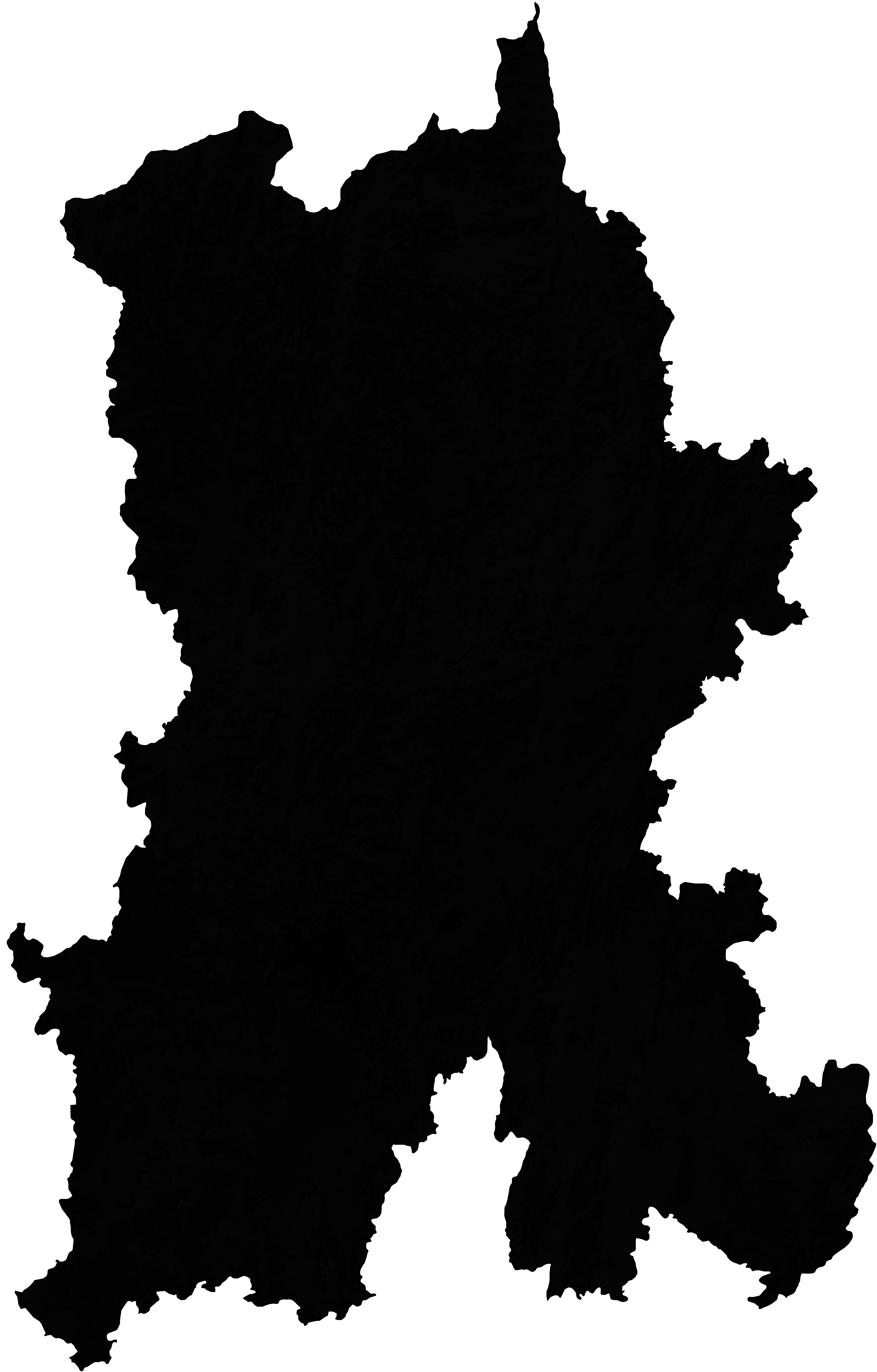

Supplement: S1 File — (ZIP) [file pone.0294462.s001.zip › S1-dataset/LUC-KM-2010.tif]

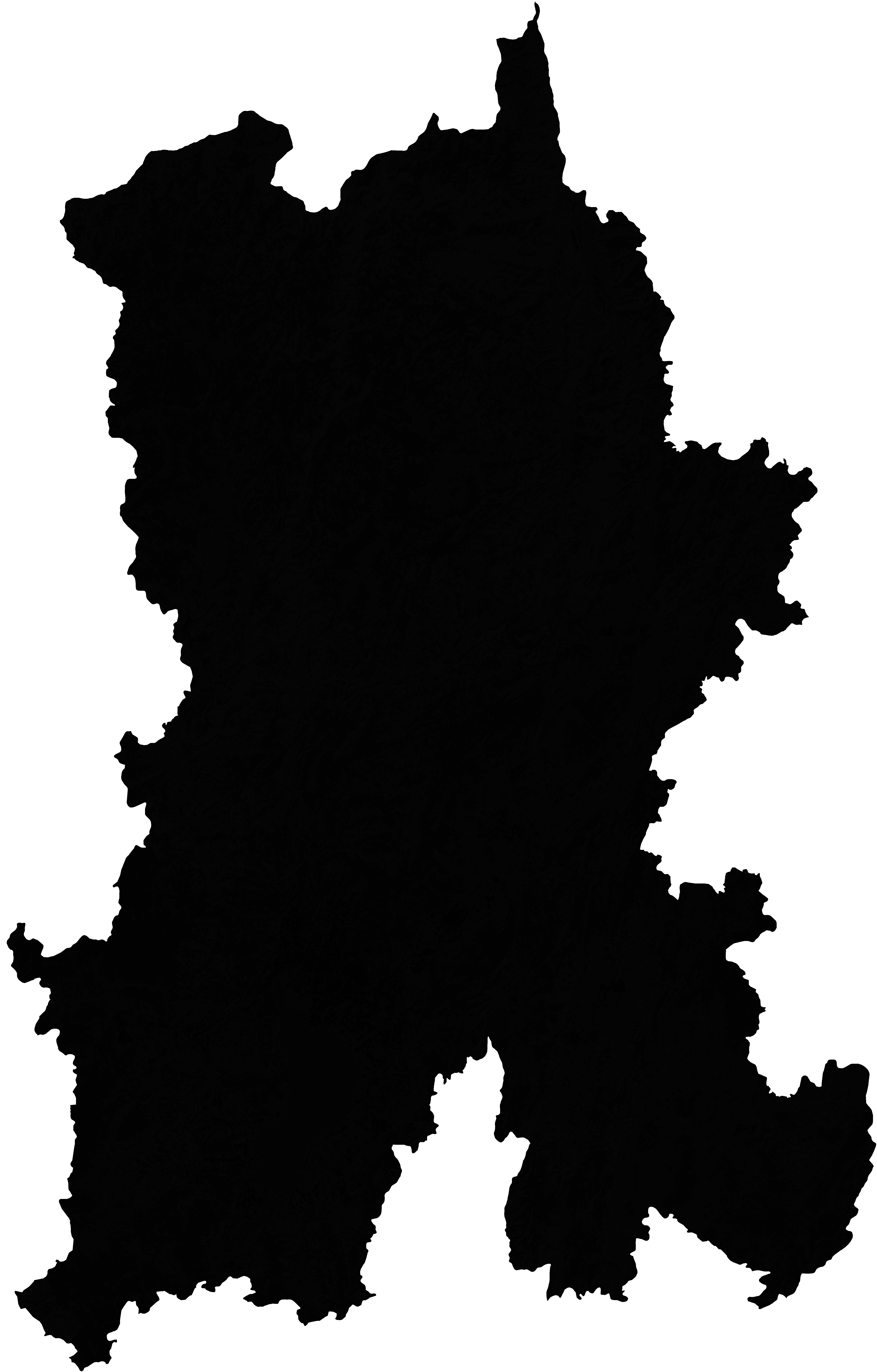

Supplement: S1 File — (ZIP) [file pone.0294462.s001.zip › S1-dataset/LUC-KM-2015.tif]

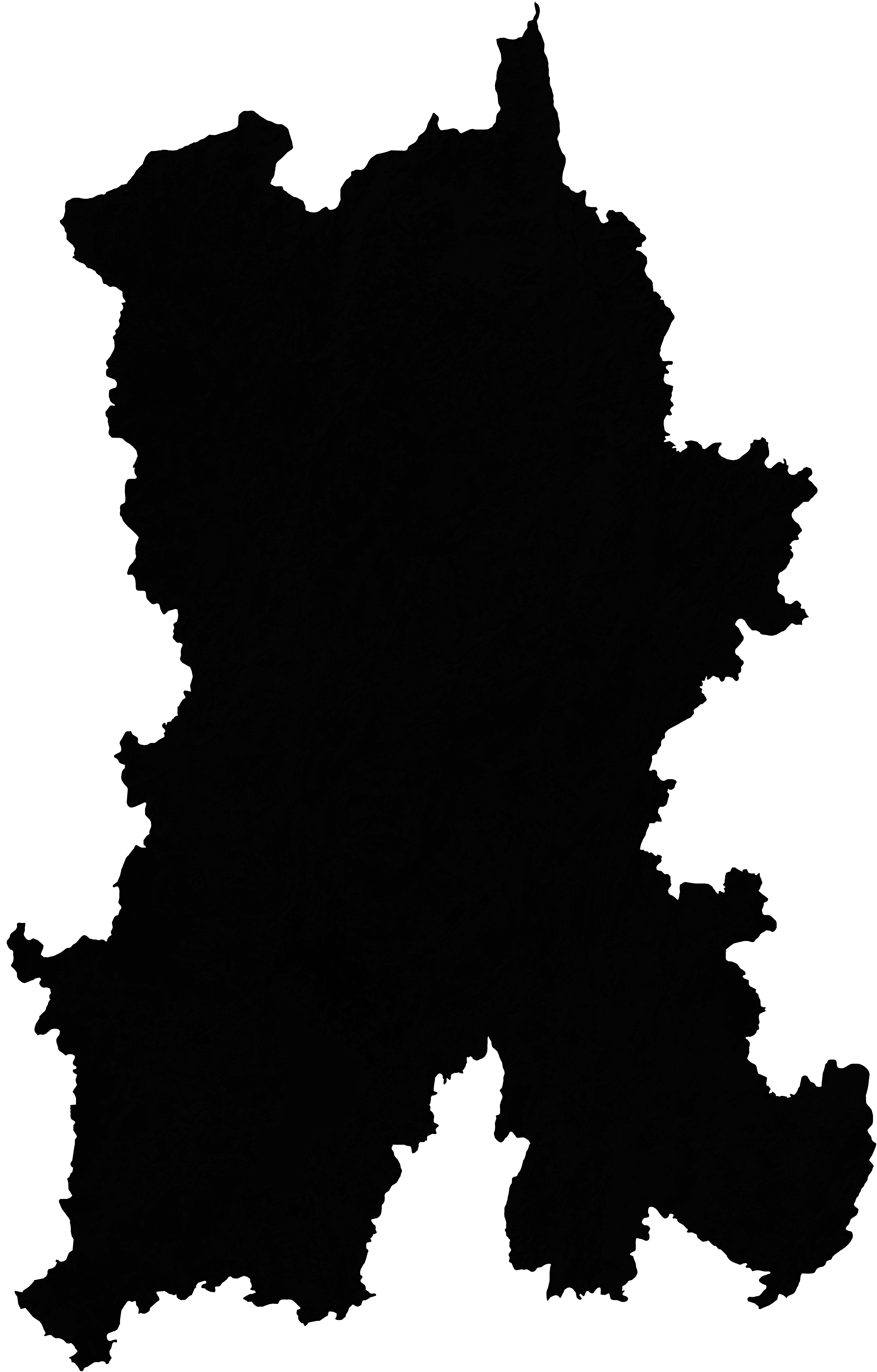

Supplement: S1 File — (ZIP) [file pone.0294462.s001.zip › S1-dataset/LUC-KM-2020.tif]
